# Assessment of Hydrothermal Treatment Effects on Coir Fibers for Incorporation into Polyurethane Matrix Biocomposites Derived from Castor Oil

**DOI:** 10.3390/polym15234614

**Published:** 2023-12-04

**Authors:** Mayara de Oliveira Camillo, Bárbara Maria Mateus Gonçalves, Veronica Scarpini Candido, Luciano Da Costa Dias, Jordão Cabral Moulin, Sergio Neves Monteiro, Michel Picanço Oliveira

**Affiliations:** 1 Forest and Wood Sciences Department, Federal University of Espírito Santo, Jeronimo Monteiro 29550-000, ES, Brazil; maycamillo@gmail.com (M.d.O.C.); barbarammateus@hotmail.com (B.M.M.G.); lucianolcd@hotmail.com (L.D.C.D.); jordao.moulin@ufes.br (J.C.M.); 2 Materials Science and Engineering Program, Federal University of Pará, Ananindeua 67140-709, PA, Brazil; scarpini@ufpa.br; 3 Materials Science Program, Military Institute of Engineering—IME, Praça General Tibúrcio 80, Urca, Rio de Janeiro 22290-270, RJ, Brazil

**Keywords:** composites, natural coir fibers, lignocellulosic fibers, polymeric, PU, mechanical properties

## Abstract

The incorporation of natural lignocellulosic fibers as reinforcements in polymer composites has witnessed significant growth due to their biodegradability, cost-effectiveness, and mechanical properties. This study aims to evaluate castor-oil-based polyurethane (COPU), incorporating different contents of coconut coir fibers, 5, 10, and 15 wt%. The investigation includes analysis of the physical, mechanical, and microstructural properties of these composites. Additionally, this study evaluates the influence of hydrothermal treatment on the fibers, conducted at 120 °C and 98 kPa for 30 min, on the biocomposites’ properties. Both coir fibers (CFs) and hydrothermal-treated coir fibers (HTCFs) were subjected to comprehensive characterization, including lignocellulosic composition analysis, scanning electron microscopy (SEM), Fourier transform infrared spectroscopy (FTIR), X-ray diffraction (XRD), and thermogravimetric analysis (TGA). The biocomposites were subjected to water absorption analysis, bending tests, XRD, SEM, FTIR, and TGA. The results indicate that the 30 min hydrothermal treatment reduces the extractive content, enhancing the interfacial adhesion between the fiber and the matrix, as evidenced by SEM. Notably, the composite containing 5 wt% CF exhibits a reduced water absorption, approaching the level observed in pure COPU. The inclusion of 15 wt% HTCF results in a remarkable improvement in the composite’s flexural strength (100%), elastic modulus (98%), and toughness (280%) compared to neat COPU. TGA highlights that incorporating CFs into the COPU matrix enhances the material’s thermal stability, allowing it to withstand temperatures of up to 500 °C. These findings underscore the potential of CFs as a ductile, lightweight, and cost-effective reinforcement in COPU matrix biocomposites, particularly for engineering applications.

## 1. Introduction

Polymers find extensive applications across various industrial sectors, including the packaging, automotive, agriculture, and food sectors [1]. The imperative to develop cost-effective and biodegradable materials has prompted industries to explore renewable natural resources [2]. In the pursuit of enhancements, numerous studies have investigated the integration of plant fibers as reinforcements in polymer matrix composites [3].

Vegetable fibers, also known as natural lignocellulosic fibers (NLFs), exhibit a range of desirable properties, including specific strength, low density, cost-effectiveness, favorable thermal characteristics, biodegradability, specific stiffness, and excellent flexural and tensile strength [4,5,6], which make our composite material highly versatile. Additionally, the incorporation of fibers, such as bamboo [7,8,9], coconut (coir) [10,11,12], banana [13,14,15], linen [16,17,18], sisal [19,20,21], and jute [21,22,23], enhances its structural integrity, making it suitable for diverse applications, including the production of plastic wood. In contrast to synthetic fibers, like carbon, steel, glass, and polypropylene, which entail substantial emissions of pollutants during production, NLFs are renewable and demand significantly less energy in their manufacturing processes [4,24].

Coir fiber (CF), extracted from the mesocarp of the coconut (*Cocos nucifera* L.), is characterized by its remarkable rigidity, withstanding elongation at breakage 4 to 6 times greater than other NLFs [24,25]. Its chemical composition comprises 26.6% cellulose, 21% hemicellulose, 29.4% lignin, 27.7% pectin, and 8.3% other components, including ash and extractives [26].

However, despite their outstanding characteristics, natural lignocellulosic fibers (NLFs) display properties that can hinder their utility as reinforcements in polymer composites. It is imperative to address these limitations, especially in the context of applications like plastic wood, where the overall composite properties are crucial. These limitations include poor surface adhesion to the matrix, low resistance to humidity, and susceptibility to microbial attacks [26]. NLFs are inherently hydrophilic, which poses compatibility issues with hydrophobic polymeric matrices. This disparity results in reduced interfacial bonding between the two materials, ultimately compromising the mechanical and thermal performance of the composite [27]. To address these challenges, various treatment methods have been developed to enhance and modify the chemical, physical, or morphological properties of fibers, facilitating improved adhesion to the hydrophobic polymer matrix [27]. These treatment approaches fall into three primary categories: chemical treatments [28,29], biological treatments [30,31], and physical treatments [32,33].

Hydrothermal treatment, a type of physical treatment, alters the fiber’s structure without affecting its chemical composition. This method involves subjecting the fibers to distilled water at elevated temperatures inside an autoclave, under steam pressure [2]. It is a treatment that inflicts minimal damage on the fibers, without the use of chemical solvents, minimizing weight losses of lignin and cellulose. In composite materials, the primary purpose of hydrothermal treatment is the removal of surface extractives [34]. Moura et al. [35] examined the effects of hydrothermal treatment at 80 °C on CFs intended for use as reinforcement in a polyhydroxybutyrate (PHB) polymer matrix. The results demonstrated that this treatment effectively removed impurities, such as waxes, leading to enhanced interfacial adhesion between the NLF and polymer matrix. The presence of fibers in the matrix also contributed to improved thermal stability in the composite.

Polyurethane (PU) stands out as one of the most versatile polymers due to its wide range of reagents that can be employed in its synthesis [36]. Depending on its processing, PU can exhibit thermoplastic, elastomeric, or thermosetting behavior, yielding various final forms, such as foams, films, and rubbers [37]. Among the diverse raw materials used for its production, castor-oil-based polyurethane (COPU), a natural polyol, is noteworthy for its commercial availability, which is attributed to its primary constituent, the ricinoleic acid [37]. This acid enables the synthesis of PU through the reaction between the –OH group in its structure and the –NCO group in diisocyanates [38]. In comparison to petroleum-derived PUs, vegetable-based PUs are less environmentally aggressive and exhibit accelerated degradation [39]. To enhance the thermal, physical, and mechanical properties of this polymer, fibers are introduced into the matrix to form polymer biocomposites [40].

Concerning the application of this material, it is important to highlight that polymeric composites reinforced with CFs are used as substitutes for wood in the so-called plastic woods and can be applied in civil construction, such as in ceilings and coatings, as well as in decks and laminated floors [41].

The primary objective of this study was to formulate biocomposites utilizing both hydrothermally treated (HTCF) and untreated CFs within a natural-origin COPU resin. The investigation involved the systematic assessment of biocomposites’ properties, such as the proportion of CFs, both in their natural state and after hydrothermal treatment. The CF content was incrementally increased. To gain insights into the mechanisms governing fiber/matrix interactions, this study encompassed a comprehensive examination of the chemical, structural, and mechanical characteristics of the CFs. Additionally, the morphological and physical attributes of the biocomposites and their interactions with COPU were thoroughly analyzed.

## 2. Materials and Methods

### 2.1. Materials

The CFs (*Cocos nucifera* L.) were obtained from a private plantation in Jerônimo Monteiro, located in the state of Espírito Santo, Brazil. The coconuts were manually opened, and each fiber was meticulously extracted, cleaned, hand-extracted, and left to air-dry naturally.

The COPU utilized in this study was synthesized from a prepolymer (IMPERVEG^®^ AGT 1315 A) and a polyol (IMPERVEG^®^ AGT 1315 B), both of which were commercially supplied by IMPERVEG^®^ Polímeros Indústria e Comércio LTDA, São Paulo, Brazil.

### 2.2. Methods

#### 2.2.1. Hydrothermal Treatment of Coir Fibers

CFs underwent a hydrothermal treatment as follows. CFs were randomly chosen and placed in a Phoenix vertical autoclave (Araraquara, Brazil) containing distilled water. The treatment was conducted for a duration of 30 min at a temperature of 120 °C and a pressure of 98 kPa. After the hydrothermal treatment, the fibers were carefully removed from the autoclave and subjected to drying. They were placed in an oven set at 103 °C for a period of 12 h to ensure thorough drying. These treated CFs were denoted as HTCFs.

#### 2.2.2. Manufacturing of Composites

CF/COPU composites were manufactured with 0, 5, 10, and 15 wt% treated and untreated fibers. First, 46% of the prepolymer synthetized from castor-oil-based polyol with diphenylmethane diisocyanate was manually mixed with 54% of polyol, also derived from castor oil, for 2 min and placed in a desiccator coupled with a pump vacuum for 1 min. Previously, these components were oven-dried for 1 h at 100 °C to reduce the moisture content of the resins. After mixing, a suitable amount of CF was added to obtain 5, 10, and 15 wt% in the composite. This process was made for plain CF and HTCF. The mixture formed was poured into a silicone mold with shapes and dimensions defined by the ASTM D7264 standard [42] and then placed in a desiccator for 3 min to induce the formation of bubbles. Finally, the mold was placed in a compressed air reactor at 90 kPa for 72 h.

#### 2.2.3. Lignocellulosic Characterization of Coir Fibers

CFs and hydrothermally treated coir fibers (HTCFs) were selected for characterization to determine their chemical composition, specifically their cellulose, hemicellulose, and lignin contents. The moisture content was determined following an adaptation of the NBR 14929:2017 standard [43]. The extraction of components was carried out based on the NBR 14853:2010 standard [44]. Lignin analysis was conducted with modifications to the NBR 7989:2010 standard [45]. Holocellulose, cellulose, and hemicellulose contents were analyzed using Browning’s methodology, as cited by Rowell et al. [46]. Ash content analysis was performed in accordance with ASTM D1762-84 [47].

#### 2.2.4. Water Absorption

The water absorption test was conducted on the CF/COPU composites in accordance with the ASTM D570-98 standard [48]. The test specimens were initially weighed to measure their masses. Subsequently, these specimens were submerged in distilled water for a period of 24 h. After the soaking period, the specimens were removed, gently dried with a damp cloth, and reweighed. This procedure was repeated over a 7-day period.

#### 2.2.5. Bending Test

The mechanical 3-point bending test was performed using the EMIC universal testing machine, model DL1000 (São José dos Pinhais, Brazil). The test was conducted on five specimens for each composition to ensure statistical validity. The specimens utilized in the 3-point bending test adhered to the dimensions specified by the ASTM D7264 standard [42]. Each specimen measured 70 mm in length, 13 mm in width, and 13 mm in thickness.

#### 2.2.6. Fourier Transform Infrared (FTIR) Spectroscopy

The presence of molecular functional groups in the fibers and composites was verified through FTIR. The spectra were acquired using a Bruker spectrophotometer, specifically the Tensor 27 model (Billerica, MA, USA), which was equipped with a ZnSe-ATR accessory. Infrared spectra were collected with 32 scans in the range of 4000 to 600 cm^−1^. The samples were prepared by grinding them in a knife mill until the particle size was less than 60 mesh.

#### 2.2.7. Scanning Electron Microscopy (SEM)

The surfaces of CFs, HTCFs and composites were observed using secondary electron SEM on the JSM-IT200 equipment model JEOL (Tokyo, Japan).

#### 2.2.8. X-ray Diffraction (XRD)

XRD patterns were obtained using the Rigaku Mini-Flex 600 Diffractometer (Tokyo, Japan), employing Cu Kα radiation with a wavelength (λ) of 1.54 Å. The samples were subjected to scanning in the 2θ range from 5° to 70° at a scan rate of 0.05° per 2θ and a speed of 2° min^−1^. The crystallinity index quantifies the ratio of crystalline structures to amorphous structures within a material, playing a pivotal role in elucidating the molecular order and physical properties in polymers and composite materials. In this study, the crystallinity index was calculated using the methodology adapted from Maradini et al. [49].

#### 2.2.9. Thermogravimetric Analysis (TGA)

Thermogravimetric (TG) and derivative (DTG) curves of plain CFs and the resulting COPU composites were obtained using the TGA/DSC LabSys EVO equipment (Caluire, France). These analyses were conducted under an inert atmosphere, with a heating rate of 10 °C/min, spanning a temperature range from 23 to 800 °C.

### 2.3. Statistical Analysis

The analysis of variance (ANOVA) was applied to test the hypothesis of equality, together with a Tukey test with a lower significant difference for the bending test and water absorption results, both with a 95% confidence level. The results of the statistical analysis are presented in Appendix A.

## 3. Results

### 3.1. Lignocellulosic Characterization of CF and HTCF

CFs are lignocellulosic materials comprising a chemical composition that contains lignin, cellulose, hemicellulose, moisture, and extractives. The intertwining of cellulose and lignin units occurs through hemicellulose chains, resulting in a complex matrix. The presence of lignin imparts oleophilic characteristics to the fibers, whereas cellulose and hemicellulose contribute hydrophilic properties to their structures [50,51].

The following Table 1 consolidates the values obtained during the chemical characterization stages of natural green CF and after undergoing hydrothermal treatment (HTCF). Additionally, the standard deviation values for these measurements are presented, providing insights into the consistency and reliability of the obtained data.

In the current study, CF exhibited an extractive content of 17%. This value aligns with findings by Nascimento et al. [52], who reported similar extractive levels at 15%. However, Souza et al. [53] reported slightly higher values at 20%.

The lignin content in the plain CF in this study was determined to be 22%. This result contrasts with findings by Souza et al. [53], Pereira et al. [54], and Lomelí-Ramírez [55], who reported higher lignin values at 30%, 31%, and 35%, respectively.

For plain CF, the present study reported percentages of 34% for cellulose and 27% for hemicellulose. These values were in proximity to those found by Cardoso et al. [51] and Pereira et al. [54] at 35% and 36% for cellulose, respectively. However, Nascimento et al. [52] reported lower hemicellulose values at 22%, and Pereira et al. [54] reported higher values at 33%.

It is important to note that variations in the lignocellulosic composition of CFs can be expected due to factors such as plant species, soil conditions, and regional climate. These variations influence values of extractives, lignin, cellulose, hemicellulose, and ash content.

After undergoing hydrothermal treatment, HTCF, a reduction in extractives and lignin content was observed, accompanied by an increase in hemicellulose content. This trend aligns with findings by Gonçalves et al. [2], who conducted a similar hydrothermal treatment on coffee husk fibers. The reduction in extractives and the rise in hemicellulose content are attributed to improvements in mechanical properties, as observed in composites reinforced with hydrothermally treated coffee husk particles [2].

### 3.2. Water Absorption

**Figure 1:**
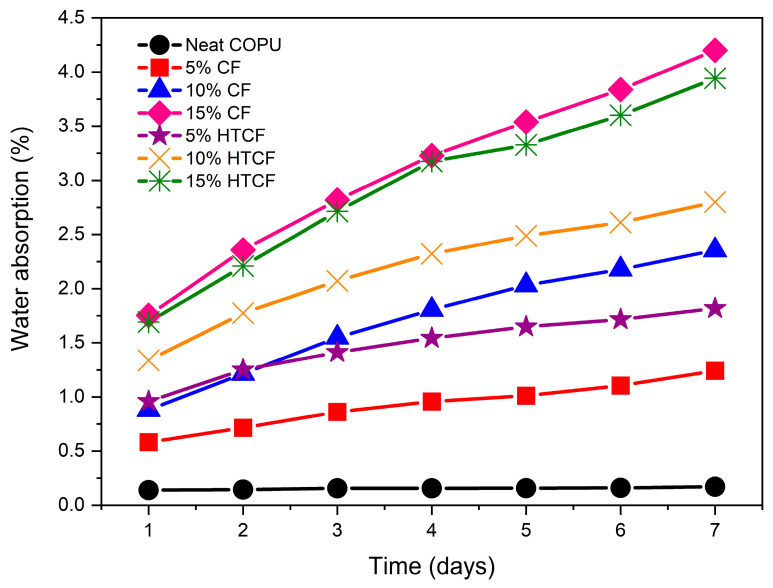
Water absorption of biocomposites.

The water absorption behavior, concerning time, is illustrated in Figure 1 for neat COPU as well as for biocomposites reinforced with 5%, 10%, and 15% of both CF and HTCF.

The incorporation of a varying CF content into the vegetable COPU resin resulted in notable distinctions among the assessed compositions, as confirmed by the analysis of variance (ANOVA) and the Tukey test, as presented in Table A1 and Table A2 in Appendix A.

Table A1 and Table A2 provide a comprehensive overview of the statistical differences observed between the compositions, further elucidating the impact of CF content on water absorption properties.

The composite containing 5 wt% CF exhibited the lowest water absorption, with a weight increase of 1.24% recorded on the seventh day. However, statistically, its variation is deemed insignificant when compared to pure COPU. Conversely, as shown in Figure 1, the composite containing 15 wt% CF displayed the highest water absorption on the final day, with a weight increase of 4.2%. Nevertheless, statistically, it absorbed a similar amount of water compared to composites with 5 wt% HTCF, 10 wt% CF and HTCF, and 15 wt% CF.

The observed increase in water absorption as the percentage of coconut fiber in the composites increases can be attributed to several factors. Primarily, it is linked to enhanced water diffusion into the voids within the composite structure, combined with the inherently hydrophilic nature of the fibers, which interact with water through defects.

Hemicelluloses play a significant role in moisture absorption within the fiber. Interestingly, as shown in Table 1, the treated fiber, HTCF, exhibited a higher hemicellulose content compared to the plain CF. This higher hemicellulose content can contribute to the increased moisture absorption observed in HTCF.

Low levels of extractives and insoluble lignin are additional factors contributing to higher water absorption levels. These components possess hydrophilic structures within the cell wall of plant fibers, facilitating water passage through fiber walls. As indicated in Table 1, treated fibers, HTCF, exhibit lower levels of extractives and insoluble lignin compared to plain CF, further promoting water penetration.

Bubbles generated during the resin curing process, attributed to exothermic reactions, also impact water absorption. While water is produced during curing, it remains insoluble in the cured resin, leading to its evaporation and the formation of bubbles. Findings from comparative studies by Faria et al. [56,57] provide additional insights. These studies on PU matrix composites with CFs revealed that the increase in fiber content did not result in significant differences in water absorption between the compositions. However, it is noteworthy that the composite with the highest fiber content demonstrated the highest water absorption, reinforcing the observed trends.

In the case of treatment, the comparison between treated and untreated composites with the same proportions of CFs did not show a reduction in water absorption. Nevertheless, it is important to highlight that the absorption values reached a maximum of 4%, which is exceptionally low. This low water absorption rate suggests the viability of using these biocomposites in various environments, demonstrating their potential for practical applications.

### 3.3. Bending Test for Flexural Properties

**Figure 2:**
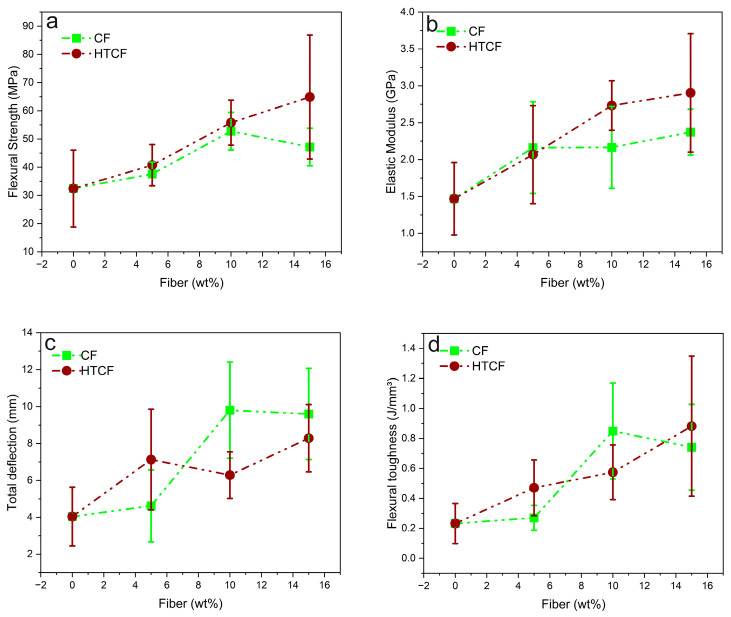
Percentage variation in CF and HTCF in the biocomposites vs. flexural properties: flexural strength (**a**); elastic modulus (**b**); total deflection (**c**); flexural toughness (**d**).

Figure 2 illustrates graphs representing key flexural properties, including the flexural strength, modulus of elasticity, deflection, and toughness, for pure COPU and composites reinforced with varying percentages (5%, 10%, and 15 wt%) of both CF and HTCF.

Figure 2a highlights that the incorporation of 15 wt% HTCF into the COPU matrix resulted in significantly greater flexural strength. However, a deeper analysis through the ANOVA and the Tukey test, as presented in Table A3 and Table A4, respectively, reveals that the composites with 10 wt% and 15 wt% treated fiber do not exhibit statistically significant differences from each other. Nevertheless, both compositions differ significantly from the others.

Table A3 and Table A4 provide a detailed breakdown of the statistical analysis, shedding light on the significant enhancements in flexural strength. When compared to pure COPU, the addition of 10 wt% and 15 wt% HTCF resulted in impressive increases in resistance of 72% and 100%, respectively.

Findings by Fiorelli et al. [58] also align with these observations. Their study involved the production of agglomerates using polyurethane adhesive derived from castor oil and urea-formaldehyde, along with natural CF. Then, a flexural test demonstrated a 12% increase in flexural strength (modulus of rupture) for CF panels with COPU resin compared to CF panels and urea-formaldehyde resin. This supports the notion that the COPU matrix with CF exhibits favorable interaction and resistance.

Figure 2b suggests that incorporating 15 wt% HTCF results in biocomposites with superior stiffness in terms of the modulus of elasticity. Nevertheless, further statistical analysis, as presented in Table A5 and Table A6, unveils that those biocomposites with 10% and 15 wt% HTCF significantly differ from the remaining compositions. As such, the addition of 10 wt% and 15 wt% HTCF provided a notable increase in the elastic modulus, with improvements of 86% and 98%, respectively, compared to pure COPU.

These results highlight the substantial enhancements in both flexural strength and modulus of elasticity achieved by incorporating HTCF into the COPU matrix. The findings are in line with the observations made by Fiorelli et al. [56] where CF panels with COPU resin exhibited an increased flexural strength and modulus of elasticity compared to panels produced with urea-formaldehyde resin.

Figure 2c illustrates that the biocomposites with 10 wt% CF exhibit greater deformation within their structure. However, a more comprehensive statistical analysis, as presented in Table A7 and Table A8, reveals that biocomposites reinforced with 10 and 15 wt% CF have a significantly increased bending deflection, with improvements of 143% and 138%, respectively, compared to other compositions.

Figure 2d demonstrates that the composite with 15 wt% HTCF exhibits greater flexural toughness. However, a detailed statistical analysis, as presented in Table A9 and Table A10, reveals that composites with 10 wt% CF and 15 wt% HTCF are statistically equal and differ significantly from the other compositions.

Compared to pure COPU, the biocomposites with 10 wt% CF achieved a remarkable 270% increase in toughness, while the biocomposites with 15 wt% HTCF showed an even greater improvement, with their toughness increasing by 283%.

### 3.4. Fourier Transform Infrared Spectroscopy (FTIR)

**Figure 3:**
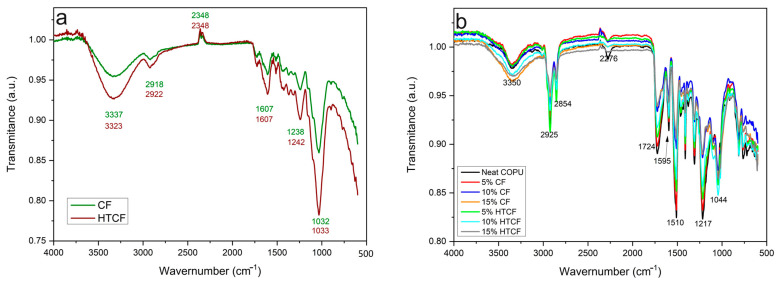
FTIR Spectra: (**a**) displays the FTIR spectra for the fibers and (**b**) presents the spectra for the biocomposites.

FTIR was employed as a key technique for the structural characterization of CF and HTCF and the composites reinforced with these fibers. The FTIR spectra for both the fibers and the composites are presented in Figure 3.

Analyzing the FTIR spectrum of plain CF in Figure 3a, intense absorption was observed at 3337 cm^−1^, indicating the axial deformation of the hydroxyl groups (OH) in cellulose. Additionally, two absorption bands within the range of 2918 to 2348 cm^−1^ represent asymmetric CH_2_ groups of extractives, alkyl and aliphatic groups of cellulose, the methyl group in hemicellulose, and the methoxy group in lignin. A characteristic band at 1607 cm^−1^ corresponds to the stretching of the C=C double bond in aromatic lignin compounds. Bands between 1500 and 1238 cm^−1^ are indicative of CH deformation in phenolic groups derived from lignin and aromatic groups, as well as the stretching of CO bonds; arylmethyl esters in lignin; and the presence of acetyl groups, carboxylic acids, and esters. The prominent absorption band at 1032 cm^−1^ is attributed to the deformation of OH groups and the stretching of C-O bonds present in polysaccharides [2,59,60].

The HTCF spectrum in Figure 3a exhibits increased absorption at 3337 cm^−1^ comparable to that of CF, which is associated with the decrease in lignin content and increase in hemicellulose content, as indicated in Table 1. This effect is consistent with the findings of Golçalves et al. [2], who conducted hydrothermal treatment on coffee husks, resulting in the enhanced removal of extractive compounds and hemicellulose dissolution [2].

In the spectra in Figure 3b, a band at 3350 cm^−1^ corresponds to the stretching of -OH bonds in both the fiber and the COPU matrix. Notably, as the fiber content increases, this band becomes broader and slightly shifts to the right, indicating interaction between the fiber and the matrix. Additionally, a reduction in the intensity of the isocyanate group peak at 2276 cm^−1^ indicates the occurrence of bonding between the urethane polymer and the coconut fiber. This chemical interaction involves the free isocyanate groups in the matrix reacting with the free hydroxyl groups in the fibers, leading to increased fiber–matrix adhesion at the interface [61,62].

In the range of 1724 to 1044 cm^−1^, in Figure 3b it is evident that the spectrum of composites with 5 wt% fiber closely resembles that of pure COPU due to the higher polymer content. Conversely, composites with 15 wt% CF and HTCF exhibit a spectrum resembling that of the plain CF spectrum in Figure 3a. This similarity in absorption intensity further validates the effective interaction between the fiber and the matrix (as observed in the peak at 3350 cm^−1^) [61].

### 3.5. Scanning Electron Microscopy (SEM)

**Figure 4:**
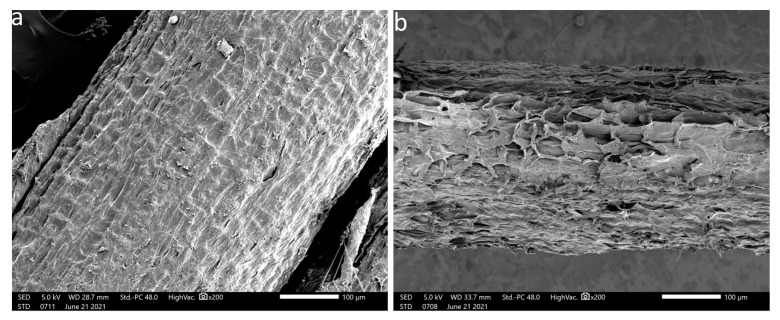
SEM Images of coir fibers: CF (**a**); HTCF (**b**).

SEM was employed to observe the morphology of coir fibers both before and after the hydrothermal treatment. These observations are visually presented in Figure 4.

In Figure 4a, it is evident that impurities, wax, and extractives cover the fiber’s surface, resulting in a relatively smooth and uniform appearance. After subjecting the fibers to hydrothermal treatment, as depicted in Figure 4b, a notable transformation in the fiber’s surface can be observed. It becomes rough and heterogeneous, indicating the partial removal of these amorphous components. This structural change aligns with findings from previous studies [35,52].

The surface properties of fibers, including their chemical composition, size, shape, and roughness, play a pivotal role in determining the quality of interfacial adhesion between the fiber and the polymer matrix. This adhesion is a critical factor influencing the overall mechanical performance of the composite.

A rough and dispersed fiber surface within the matrix offers a higher potential for physical anchoring between the fiber and the polymer matrix. Enhanced physical interaction can lead to an improved load transfer and, consequently, better mechanical properties of the composite.

**Figure 5:**
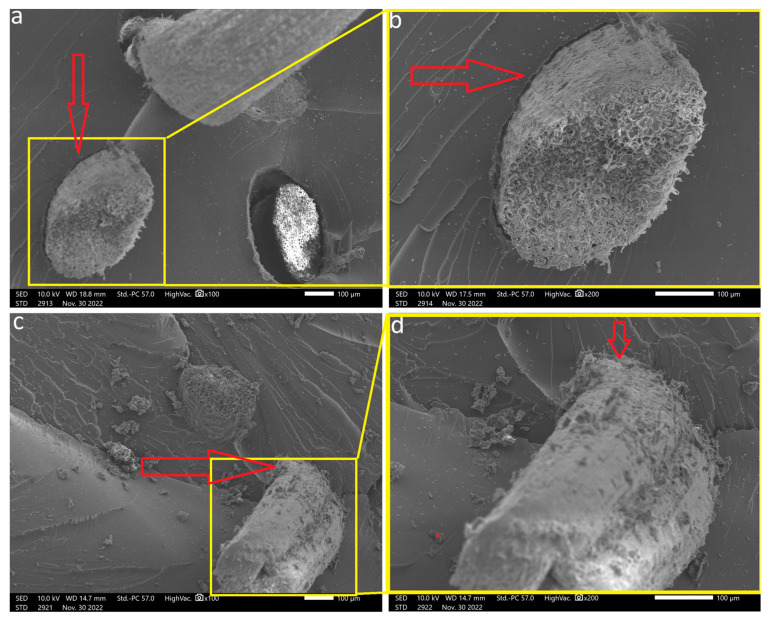
SEM images of biocomposites: (**a**,**b**) depict the interfacial bond between the COPU matrix and CF fibers at magnifications of 100× and 200×, respectively. (**c**,**d**) illustrate the interfacial bond between the COPU matrix and HTCF fibers at magnifications of 100× and 200×. Arrows indicate interface details.

Figure 5 provides a visual representation of the interaction between the CFs and the COPU matrix, captured through SEM. Notably, it becomes evident that hydrothermally treated coir fibers (Figure 5c,d) can exhibit a superior fixation within the matrix compared to their natural counterparts (Figure 5a,b). This enhanced fixation suggests that the treatment process contributes to better adhesion at the fiber–matrix interface [35].

**Figure 6:**
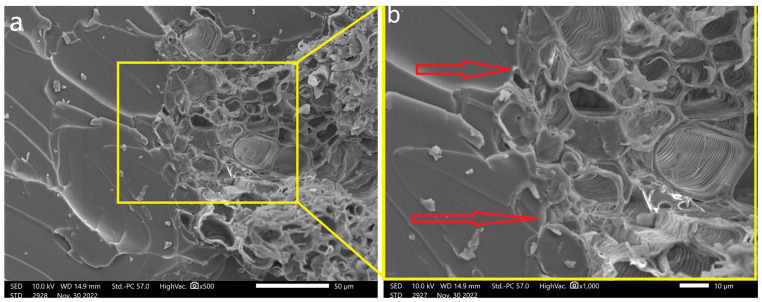
The interface between the COPU matrix and ECF was examined using SEM at magnifications of 500× (**a**) and 1000× (**b**). Arrows indicate connection points, emphasizing the continuity between the matrix and the fibers.

The observed differences in fixation and surface adhesion between natural and treated fibers reveals the importance of fiber surface modification techniques, such as hydrothermal treatment, in enhancing the overall mechanical performance of biocomposites. Figure 6, captured through SEM, shows a point of surface adhesion between the HTCF and COPU.

### 3.6. X-ray Diffraction (XRD) Analysis and Crystallinity Index

**Figure 7:**
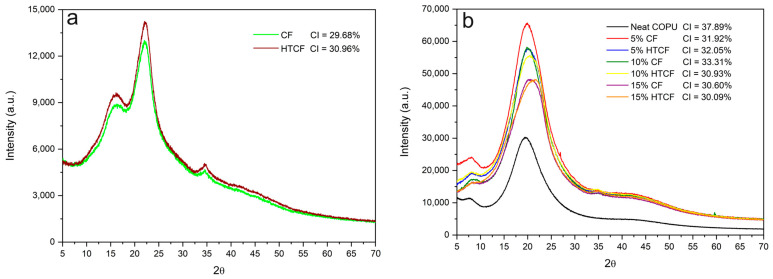
X-ray diffraction of coir fibers (**a**); X-ray diffraction of biocomposites (**b**).

XRD analysis serves as a valuable tool for assessing various aspects of the crystal structure within the materials. In this study, it was employed to determine the crystallinity index of the examined components, including plain CF and HTCF, neat COPU, and the resulting biocomposites. The crystallinity index values of CF, HTCF, COPU, and the biocomposites are shown in Figure 7. These values provide insights into the degree of crystalline order within the materials. Figure 7a showcases the XRD patterns of the fibers, shedding light on their crystalline structures and any variations resulting from the hydrothermal treatment. Figure 7b presents the XRD patterns of the biocomposites, revealing how the incorporation of fibers influences the crystalline characteristics of the resulting materials.

The crystallinity index is a key parameter, as it reflects the extent of crystalline regions within the materials, which can significantly impact their mechanical and thermal properties. A higher crystallinity index often corresponds to enhanced stiffness and thermal stability.

In Figure 7a, the crystallinity index comparison between natural and treated fibers reveals a significant increase in the crystallinity index of the HTCF. This observation aligns with the understanding that the treatment effectively removed amorphous components from the fiber structure, such as lignin and extractives [52,63].

Nascimento et al. [52] found a crystallinity index of 46.5% for raw coconut fibers and 51.1% for fibers modified using the formosolv process. Golçalves et al. [63] conducted research with natural CF and subjected it to hydrothermal treatment with 2.5% sodium hydroxide at 180 °C for 30 min. Their findings showed that the natural fiber had a crystallinity index of 34.34%, while the treated fiber exhibited a higher crystallinity index of 56.38%. This again emphasizes the impact of treatment in increasing the crystallinity index by removing amorphous structures.

Comparing the crystallinity index of the composites with the COPU resin (Figure 7b), it becomes apparent that the incorporation of fibers does not lead to a significant change in the crystallinity of the composites. In COPU, the crystallinity is primarily influenced by the degree of phase separation between the soft segments (polyols) and the hard segments (diisocyanates and chain extenders). When reinforcement materials are introduced into the matrix, microphase separation is enhanced, as evidenced by the XRD spectra of pure COPU and the composites in Figure 7b [64].

### 3.7. Thermogravimetric Analysis

**Figure 8:**
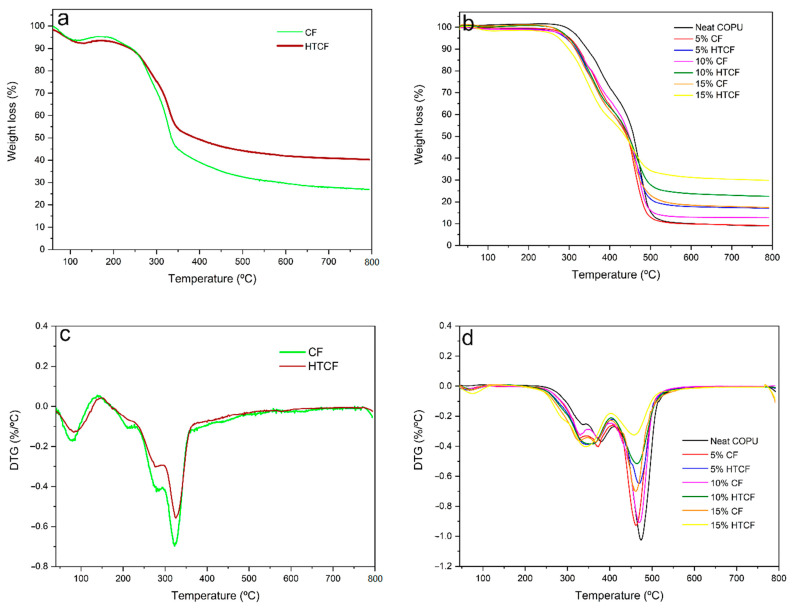
Thermogravimetric analysis of fiber (**a**) and biocomposites (**b**); differential thermogravimetric of fiber (**c**) and biocomposites (**d**).

In Figure 8, the thermogravimetric TG and derivative DTG curves for CF, HTCF, COPU, and biocomposites are displayed. The TGA curves reveal the weight loss of the samples as the temperature increases, while the DTG curves provide detailed information about the degradation process.

In Figure 8a,c, both CF and HTCF exhibit an initial degradation temperature (T_0_) of approximately 230 °C and a final degradation temperature (T_f_) of around 345 °C. However, CF experiences a higher weight loss during the process (45% by weight) compared to HTCF (35% by weight). The initial weight loss at around 70 °C for CF and 60 °C for HTCF is attributed to the dehydration of the fibers, with HTCF releasing moisture at a slightly lower temperature due to its higher porosity resulting from the hydrothermal treatment.

In the DTG curves, two stages of decomposition can be observed, corresponding to the degradation of hemicellulose and cellulose, followed by the final decomposition of lignin at approximately 600 °C. The maximum temperatures for hemicellulose degradation are 278 °C for HTCF and 283 °C for CF, while cellulose decomposition occurs at 322 °C for CF and 326 °C for HTCF. These results indicate that hydrothermal treatment slightly increases the thermal resistance of the fibers, as evidenced by the higher temperature values for the degradation of hemicellulose and cellulose.

In Figure 8b,d, the TG and DTG curves for neat COPU and its biocomposites are presented. The thermal degradation process of COPU is complex due to its chemical composition, which includes urethane bonds, urea, ester, ether, aromatic compounds, and components from the polyol and isocyanate sources. The degradation of COPU involves various processes, including the breaking of urethane bonds, dissociation of urethane into isocyanate and alcohol, formation of primary and secondary amines, olefin production, carbon dioxide generation, and transesterification-type reactions.

There is a notable difference in the initial degradation temperature (T_i_) between neat COPU (approximately 282 °C) and biocomposites (around 255 °C). The lower Ti in biocomposites is attributed to the presence of coir fibers, which release moisture into the environment. Despite this difference, the final degradation temperature (T_f_) is similar for all samples, approximately 500 °C.

Biocomposites with a higher fiber content, such as 15 wt% CF, exhibit behavior in the curve that closely resembles plain fibers. This similarity arises from the significant proportion of fibers in the composite, which provides protection against matrix degradation and results in less mass loss during the process.

## 4. Summary and Conclusions

This study delved into the utilization of coconut husk coir fiber (CF), an abundant waste material in coastal regions of Brazil, as a reinforcement in castor oil-based polyurethane (COPU) composites. Several key conclusions were drawn from the research:

Hydrothermal treatment (HTCF) effectively eliminated impurities and waxes from the fiber’s surface, enhancing its interaction with the COPU matrix. SEM analysis confirmed improved fiber–matrix adhesion, while FTIR analysis revealed distinct absorption bands between treated and untreated fibers.

Water absorption analysis demonstrated that composites with 5 wt% CF exhibited minimal water absorption, comparable to pure COPU, indicating that the incorporation of CF did not significantly increase water absorption. Composites with 10% and 15% HTCF displayed substantial enhancements in flexural strength. Particularly, the 15% HTCF composite exhibited a remarkable 100% increase in flexural strength, a 98% increase in elastic modulus, and a 280% increase in toughness.

XRD analysis revealed a higher crystallinity index in HTCF compared to CF, indicating the removal of amorphous impurities and a more crystalline structure. TGA demonstrated that coconut fiber improved the thermal stability of the polyurethane matrix, enabling it to withstand temperatures up to 500 °C.

In terms of practical applications, the 30 min hydrothermal treatment at 120 °C effectively removed amorphous components, enhancing the composite’s mechanical properties. This positions CF as a promising and cost-effective waste material for sustainable composites with a COPU matrix. Particularly noteworthy is its potential as a substitute for plastic wood in civil engineering applications, including flooring, ceilings, and domestic furniture. This innovative application contributes significantly to the development of eco-friendly materials and the utilization of abundant waste resources.

## Figures and Tables

**Table 1:** Results of the chemical characterization of CF and HTCF.

	CF	HTCF
Extractives (%)	17.0 ± 1.0	15.0 ± 0.5
Lignin (%)	22.0 ± 0.5	18.0 ± 1.0
Cellulose (%)	34.0 ± 0.5	33.0 ± 1.5
Hemicellulose (%)	27.0 ± 2.0	34.0 ± 2.0
Ashes (%)	1.0 ± 0.1	0.8 ± 0.1

## Data Availability

The data is contained within the article.

## Appendix A

**Table A1:** ANOVA of average water absorption.

Variation Causes	DF	Sum of Squares	Mean Square	F (Calc.)	F Critical (Tab.)
Treatments	6	49.0352	8.17254	31.23	5.676 × 10^−14^ *
Residue	42	10.9927	0.26173		
Total	48	60.0279			

* Critical F value smaller than the calculated F value.

**Table A2:** Tukey’s pairwise comparisons for average water absorption.

Fiber Content (wt%)	Neat COPU	5 CF	10 CF	15 CF	5 HTCF	10 HTCF	15 HTCF
Neat COPU	-	0.09562	0.000153 *	0.000138 *	0.000465 *	0.000138 *	0.000138 *
5 CF	3.985	-	0.080700	0.000138 *	0.416900	0.000710 *	0.000138 *
10 CF	8.076	4.09100	-	0.000186 *	0.975000	0.574200	0.001046 *
15 CF	15.700	11.72000	7.627000	-	0.000140 *	0.012830 *	0.974200
5 HTCF	6.844	2.85900	1.232000	8.859000	-	0.139400	0.000187 *
10 HTCF	10.580	6.59600	2.504000	5.123000	3.737000	-	0.111900
15 HTCF	14.460	10.48000	6.388000	1.239000	7.620000	3.883000	-

* Groups that differ.

**Table A3:** Analysis of variance for flexural strength.

Variation Causes	DF	Sum of Squares	Mean Square	F (Calc.)	F Critical (Tab.)
Treatments	6	3846.62	641.103	5.025	0.0013 *
Residue	28	3572.61	127.593		
Total	34	7419.23			

* Critical F value smaller than the calculated F value.

**Table A4:** Tukey’s pairwise comparisons for flexural strength.

Fiber Content (wt%)	Neat COPU	5 CF	10 CF	15 CF	5 HTCF	10 HTCF	15 HTCF
Neat COPU	-	0.9901	0.1013	0.4024	0.9027	0.04019 *	0.00179 *
5 CF	1.021	-	0.3674	0.8283	0.9994	0.18110	0.01081 *
10 CF	4.023	3.0030	-	0.9846	0.6318	0.99950	0.62390
15 CF	2.913	1.8920	1.1100	-	0.9698	0.88460	0.20440
5 HTCF	1.642	0.6214	2.3810	1.2710	-	0.37540	0.03114 *
10 HTCF	4.624	3.6030	0.6006	1.7110	2.9820	-	0.85890
15 HTCF	6.422	5.4020	2.3990	3.5090	4.7800	1.79800	-

* Groups that differ.

**Table A5:** Analysis of variance for modulus of elasticity.

Variation Causes	DF	Sum of Squares	Mean Square	F (Calc.)	F Critical (Tab.)
Treatments	6	6.6623	1.11039	3.484	0.01065 *
Residue	28	8.9249	0.31874		
Total	34	15.5872			

* Critical F value smaller than the calculated F value.

**Table A6:** Tukey’s pairwise comparisons for modulus of elasticity.

Fiber Content (wt%)	Neat COPU	5 CF	10 CF	15 CF	5 HTCF	10 HTCF	15 HTCF
Neat COPU	-	0.4716	0.4661	0.1876	0.6378	0.02144 *	0.006552 *
5 CF	2.746	-	1.0000	0.9968	1.0000	0.68560	0.391300
10 CF	2.759	0.01284	-	0.9970	1.0000	0.69110	0.396400
15 CF	3.576	0.83020	0.8174	-	0.9764	0.94760	0.746800
5 HTCF	2.367	0.37840	0.3912	1.2090	-	0.51910	0.258200
10 HTCF	5.003	2.25800	2.2450	1.4270	2.6360	-	0.998900
15 HTCF	5.687	2.94100	2.9280	2.1110	3.3190	0.68330	-

* Groups that differ.

**Table A7:** Analysis of variance for total deflection.

Variation Causes	DF	Sum of Squares	Mean Square	F (Calc.)	F Critical (Tab.)
Treatments	6	155.769	25.9614	5.754	0.000532 *
Residue	28	126.338	4.51206		
Total	34	282.107			

* Critical F value smaller than the calculated F value.

**Table A8:** Tukey’s pairwise comparisons for total deflection.

Fiber Content (wt%)	Neat COPU	5 CF	10 CF	15 CF	5 HTCF	10 HTCF	15 HTCF
Neat COPU	-	0.9994	0.003301 *	0.004874 *	0.2791	0.6382	0.05125
5 CF	0.6135	-	0.009785 *	0.01434 *	0.5177	0.8725	0.12830
10 CF	6.0720	5.459	-	1.00000	0.4405	0.1581	0.91270
15 CF	5.8520	5.239	0.219900	-	0.5350	0.2105	0.95510
5 HTCF	3.2530	2.639	2.820000	2.60000	-	0.9954	0.97540
10 HTCF	2.3670	1.753	3.706000	3.48600	0.8860	-	0.74910
15 HTCF	4.4720	3.858	1.601000	1.38100	1.2190	2.1050	-

* Groups that differ.

**Table A9:** Analysis of variance for flexural toughness.

Variation Causes	DF	Sum of Squares	Mean Square	F (Calc.)	F Critical (Tab.)
Treatments	6	2.09281	0.348801	4.933	0.001478 *
Residue	28	1.97977	0.070706		
Total	34	4.07258			

* Critical F value smaller than the calculated F value.

**Table A10:** Tukey’s pairwise comparisons for flexural toughness.

Fiber Content (wt%)	Neat COPU	5 CF	10 CF	15 CF	5 HTCF	10 HTCF	15 HTCF
Neat COPU	-	1	0.01554 *	0.06885	0.7877	0.4167	0.009727 *
5 CF	0.3183	-	0.02667 *	0.11050	0.8910	0.5529	0.01690 *
10 CF	5.1920	4.874	-	0.99460	0.3010	0.6611	1.00000
15 CF	4.2820	3.964	0.91020	-	0.6776	0.9514	0.979100
5 HTCF	2.0060	1.687	3.18700	2.27600	-	0.9957	0.218400
10 HTCF	2.8780	2.559	2.31400	1.40400	0.8721	-	0.541800
15 HTCF	5.4620	5.144	0.27000	1.18000	3.4560	2.5840	-

* Groups that differ.
